# Tick-borne lymphadenopathy in northeastern France: a human and vector clinical–epidemiological study

**DOI:** 10.1186/s13071-025-07078-2

**Published:** 2025-10-24

**Authors:** Jeanne Kotzyba, Elisabeth Baux, Yves Hansmann, Pierre Boyer, Benoît Jaulhac, Martin Martinot, Benoît Martha, Charlotte Kaeuffer, Thomas Bonijoly, Firouzé Bani-Sadr, Antoine Legoff, Florence Hoefler, Mathieu Blot, Loïc Bourdellon, Laurence Zilliox, Nathalie Boulanger, Benjamin Lefevre

**Affiliations:** 1 University of Lorraine, University Hospital of Nancy, Nancy, France; 2 Tick Borne Disease Reference Center of Northeastern Region, University Hospital of Nancy, Nancy, France; 3 Tick Borne Disease Reference Center of Northeastern Region, University Hospital of Strasbourg, Strasbourg, France; 4 UR3073: PHAVI: Group Borrelia, National Reference Center for Borrelia, University of Strasbourg, Strasbourg, France; 5 Hospital of Colmar, Colmar, France; 6 Hospital of Chalon-Sur-Saône, Chalon-Sur-Saône, France; 7 Hospital of Mulhouse, Mulhouse, France; 8 Hospital of Sélestat, Sélestat, France; 9 University Hospital of Reims, Reims, France; 10 Metz Private Hospital Group, Metz, France; 11 Hospital of Troyes, Troyes, France; 12 University Hospital of Dijon-Bourgogne, Dijon, France; 13 Hospital Center of Epinal, Epinal, France; 14 Inserm, INSPIIRE, University Hospital of Nancy, University of Lorraine, Nancy, France

**Keywords:** TIBOLA, DEBONEL, SENLAT, Tick-borne disease, Rickettsiosis, Inoculation eschar, *Dermacentor*

## Abstract

**Background:**

Tick-borne lymphadenopathy (TIBOLA) is a tick-borne disease transmitted by *Dermacentor* ticks and is usually caused by *Rickettsia*. In 2021, clinicians in northeastern France reported an increase in TIBOLA cases.

**Methods:**

This entomo-clinical, multicenter, retrospective, and observational study aimed to describe the evolution of the number of TIBOLA cases between 2016 and 2021 in northeastern France as well as the evolution of the *Dermacentor* tick population.

**Results:**

A total of 35 cases of TIBOLA were identified, 16 of which occurred in 2021, with clear predominance in April and May. A longitudinal study performed in the Alsace region (endemic for ticks and tick-borne diseases) revealed a peak in tick activity in 2021. A trend toward an increase in TIBOLA cases in 2021 in the northeastern region of France was observed, as was an increase in the *Dermacentor* tick population in some biotopes.

**Conclusions:**

TIBOLA appears to be an emerging disease that should be monitored, as should the *Dermacentor* population.

**Graphical Abstract:**

**Figure:**
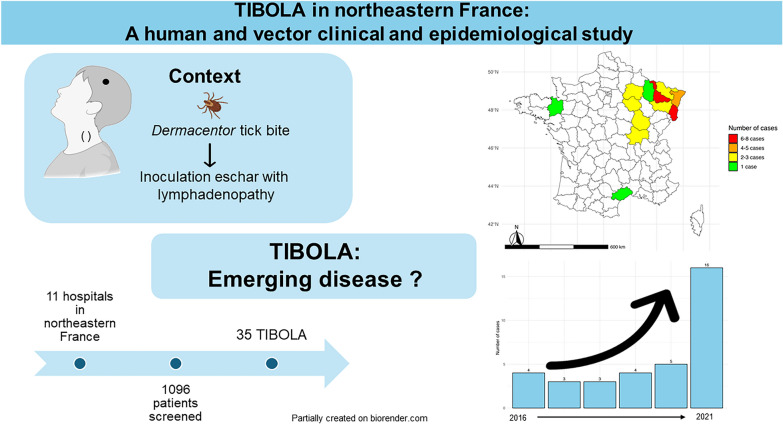

## Background

Tick borne lymphadenopathy (TIBOLA), also called *Dermacentor*-borne necrosis erythema and lymphadenopathy (DEBONEL) or scalp eschar and neck lymphadenopathy after tick bite (SENLAT), is a disease first described in Hungary in 1997 [1]. It is the second most common rickettsial disease in Europe after Mediterranean spotted fever [2]. *Rickettsia slovaca* and *R. raoultii* are the main pathogens [3]. Nevertheless, other intracellular bacteria (*Bartonella henselae* [4, 5], *Rickettsia massiliae* [6], *Candidatus Rickettsia rioja* [4], and *Francisella tularensis* [7]) have been identified. Symptoms include a necrotic inoculation eschar on the scalp and painful regional lymphadenopathies, as well as more general symptoms (fever, headache, arthromyalgia, and asthenia). This syndrome particularly affects women and children [8, 9].

Hard ticks in the *Dermacentor* genus transmit *R. slovaca* and *R. raoultii*: *Dermacentor marginatus* is found mainly around the Mediterranean area and in North Africa, and *Dermacentor reticulatus* lives mostly in the colder parts of Western Europe [8]. TIBOLA is therefore more frequent during periods of tick activity, such as spring and autumn [10].

Microbiological diagnosis is based on polymerase chain reaction (PCR), skin biopsy, swabs of the inoculation eschar or serology [11]. Unfortunately, these tests often yield false negatives [8], and the diagnosis is based on a set of arguments (tick bite on the upper part of the body, followed by the apparition of a necrotic eschar and painful regional lymphadenopathies). Systemic complications are rare, but localized alopecia may persist [4].

Since some tick-borne diseases are becoming more common in Europe (e.g., tick-borne encephalitis [12] and tularemia [13, 14]) and since 2021, clinicians in the northeastern region of France have noticed an unusual number of TIBOLA cases, documenting this clinical observation and comparing it with the presence of *Dermacentor* tick vectors in the environment is important.

The primary objective of this study was to describe the evolution of the number of TIBOLA cases between 2016 and 2021 in northeastern France as well as the evolution of the *Dermacentor* tick population. The secondary objectives were to describe the characteristics of the patients.

## Methods

### Study design and participants

In accordance with the Strengthening the Reporting of Observational Studies in Epidemiology (STROBE) recommendations, a retrospective, multicenter, and observational study was performed. A total of 18 hospitals were approached, and 11 ultimately participated. The study included patients from hospitals in Chalon-sur-Saône, Colmar, Dijon, Epinal, Metz, Mulhouse, Nancy, Reims, Sélestat, Strasbourg, and Troyes (northeastern France). Clinicians recorded information on patients with TIBOLA diagnosed between 1 January 2016 and 31 December 2021. Data collection took place between July 2022 and March 2024. Minors or adult patients who were diagnosed with TIBOLA were eligible.

Patients were identified in two complementary ways. First, clinicians from different hospitals were contacted. Furthermore, *Rickettsia* spp. serologies and PCRs performed between 2016 and 2021 in the study centers were extracted from the hospital data. From these positive or negative biological results, the patients’ medical files were consulted, and TIBOLA cases were included.

The following criteria were used to define probable and certain cases of TIBOLA:

Certain cases: compatible clinical presentation (inoculation eschar with regional lymphadenopathy, with or without general signs) and possible tick bite and microbiological evidence (positive PCR or serology for a microorganism known to be responsible for TIBOLA). *Rickettsia* serology was considered positive if the antibody titers exceeded 32 for immunoglobulin (IgM and 64 for IgG or if the IgG levels were four times higher than those in a previous serum sample [15]. The technique used for serology was specific to each center. PCR was considered positive if a pathogen responsible for TIBOLA was identified. Samples from humans (skin biopsy and swabs of the inoculation eschar) were analyzed at the National Reference Center for *Rickettsia* in Marseille [16].

Probable cases: compatible clinical presentation (inoculation eschar with regional lymphadenopathy with or without general signs) and tick bite observed without microbiological evidence.

Case definition is based on TIBOLA syndrome (inoculation eschar associated with regional lymphadenopathy, regardless of the pathogen identified (*Rickettsia*, *Francisella*)).

### Clinical data

Patient medical records were reviewed by investigators, who used a standardized form to collect various sociodemographic variables (age, sex, place of diagnosis, place of residence, urban or rural home, proximity to forests, fields, waterways, and previous tick bites). TIBOLA characteristics were collected (date of exposure, date of tick bite, date of tick removal, place of tick bite, symptoms, duration of symptoms, and incubation period). The symptom details collected were inoculation eschar (location, pain, and duration), lymphadenopathy (location, number, pain, size, and duration), fever, headache, arthromyalgia, pruritus, rash, facial edema, asthenia, and other symptoms. Biological and microbiological data were collected, including serology and PCR data for *Rickettsia* spp. and *F. tularensis* and serology data for *Borrelia*. If available, tick identification and PCR results for the tick were also reported.

Details on the antibiotic treatment (molecule, duration, and number of antibiotic courses) were also recorded. On the basis of the French recommendations [17], doxycycline and macrolides were considered appropriate, as was ciprofloxacin if *F. tularensis* was identified as the etiologic agent. Ineffective antibiotics included amoxicillin, amoxicillin/clavulanic acid, pristinamycin, and topical fusidic acid.

### Tick collection and processing

The *Borrelia* National Reference Center has been collecting *Ixodes* and *Dermacentor* ticks as part of its activity since 2014, and these data are presented here.

Questing ticks were collected monthly by dragging a white cotton cloth (1 m^2^) over vegetation as described previously [18] at four different sites in Alsace: two lowland sites (Illkirch and Dannemarie) and two mountain sites (Murbach and Niedermunster) between 2014 and 2023. The abundance of ticks by season was also investigated.

Tick identification was performed using morphology (by an entomologist), PCR, or matrix-assisted laser desorption ionization–time of flight (MALDI‒TOF) mass spectrometry targeting the cytochrome oxidase I (COI) or 16S rDNA genes as described previously [19]. PCR was performed at the *Rickettsia* National Reference Centre in Marseille, France. Tick identification was performed either in Marseille or at the *Borrelia* French National Reference Centre in Strasbourg, France.

### Statistical analysis

Patient characteristics are described as frequencies and percentages for categorical variables or medians and interquartile ranges (IQRs) for continuous variables.

## Results

**Figure 1:**
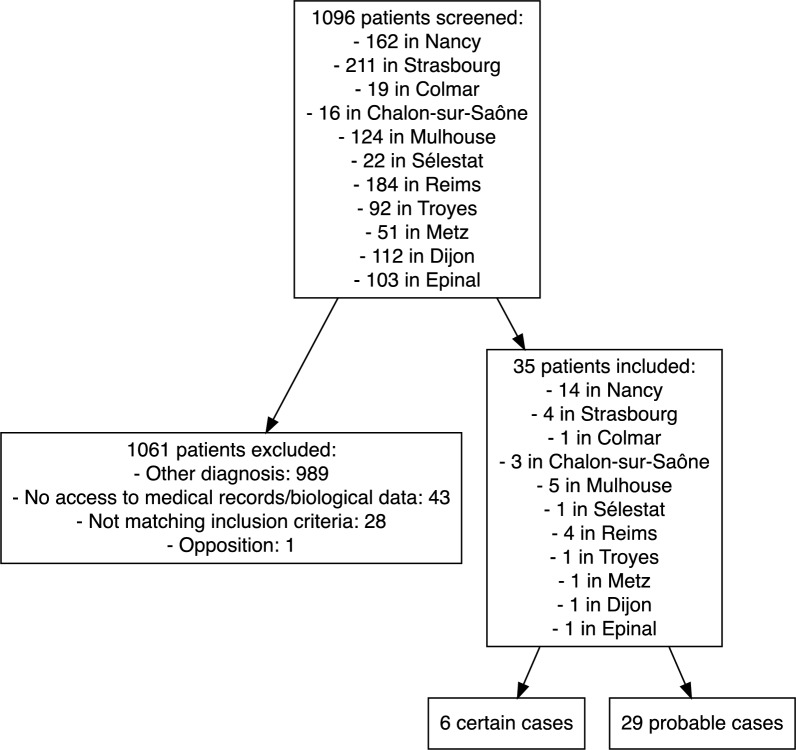
Flow chart of patients retrospectively identified as TIBOLA in northeastern France between 2016 and 2021

From *Rickettsia* spp. serologies and PCRs performed between 2016 and 2021, 1096 medical records were screened. A total of 35 certain or probable cases of TIBOLA were included, among which 6 were certain cases (17%) and 29 were probable cases (83%) (Fig. 1).

Among the certain cases, microbiological documentation was obtained by serology (*Rickettsia* in one patient, *F. tularensis* in two patients) and PCR (*Rickettsia* in four patients, *F. tularensis* in one patient). Some patients were both serologically positive and PCR positive.

**Figure 2:**
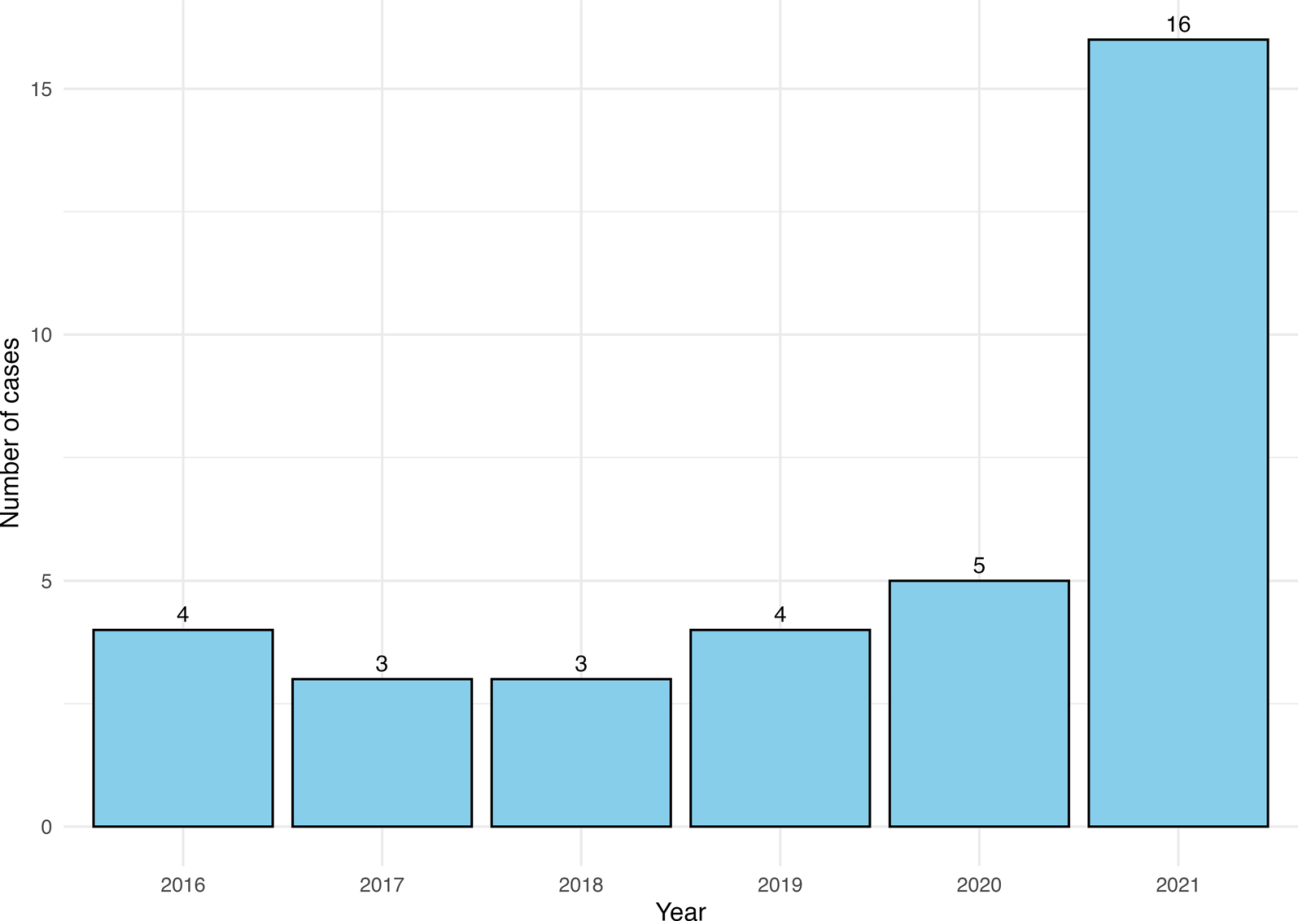
Evolution of the number of TIBOLA cases between 2016 and 2021 in northeastern France

Among the 35 patients, 16 (46%) were reported in 2021, as shown in Fig. 2, which illustrates the steady increase in the number of cases from 2016 to 2021.

### Population, epidemiology, and demography

**Figure 3:**
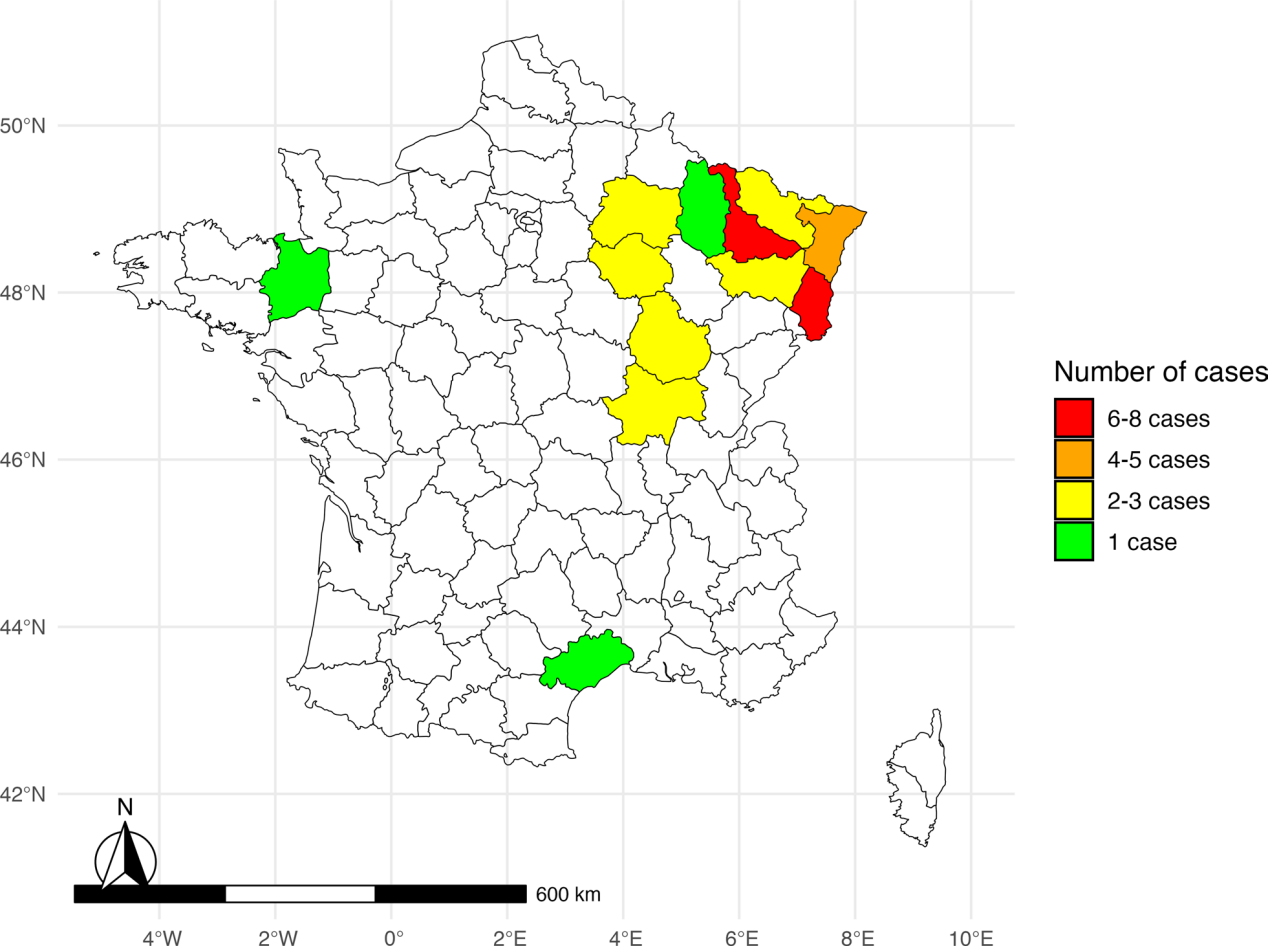
Spatial distribution of the locations in which patients affected by TIBOLA were bitten (or if unavailable, their place of residence)

**Figure 4:**
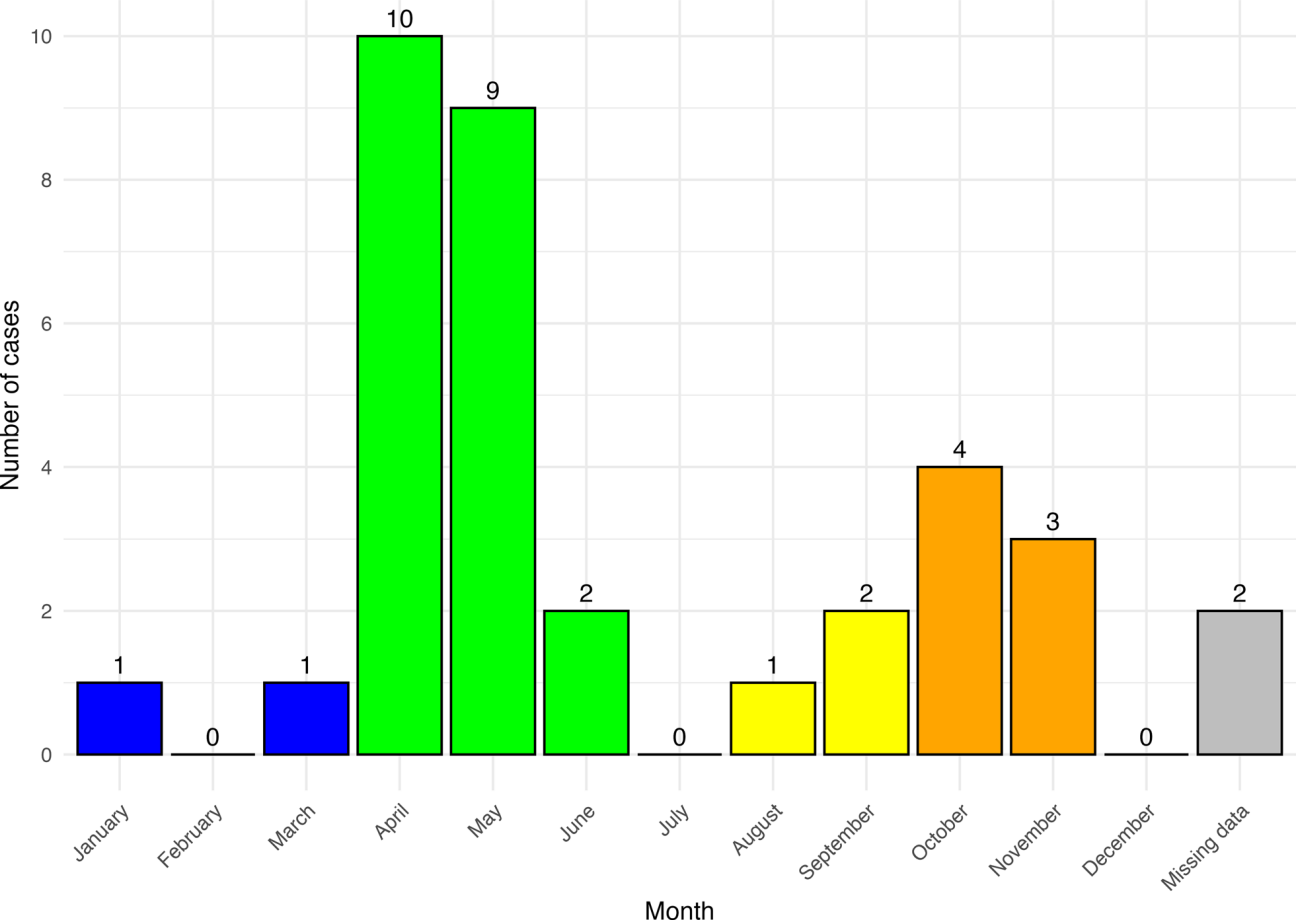
Monthly distribution of TIBOLA cases diagnosed between 2016 and 2021 in northeastern France

The study population is predominantly female (sex ratio: 4.8 women to 1 man), young, and living in rural areas. Table 1 presents the patients’ sociodemographic and exposure characteristics. The spatial distribution of the locations where patients were bitten (or if unavailable, their place of residence) is presented in Fig. 3. Concerning the monthly distribution (Fig. 4), most cases of TIBOLA occurred in April and May.

**Table 1:** Characteristics of patients retrospectively identified with TIBOLA in several hospitals in northeastern France between 2016 and 2021

Sociodemographic data	
Female, no. (%)	29 (83)
Male, no. (%)	6 (17)
Median age, years (IQR^a^)	32.5 (10–55)
< 18 years old, no. (%)	12 (34)
Median age, years (IQR)	9 (6–9)
Place of residence	
Rural, no. (%)	20 (57)
Urban, no. (%)	15 (43)
Living close to field, gardens, forests or watercourses, *N* = 20, no. (%)	18 (90)

^*a*^*IQR* interquartile range

When *N* is not precise, the denominator is the total effective rate (*N* = 35)

### Clinical characteristics

The median incubation period was 4 days (IQR 2–8 days). The clinical features and durations of symptoms of the patients are presented in Table 2.

**Table 2:** Clinical characteristics of patients retrospectively identified as having TIBOLA in several hospitals in northeastern France between 2016 and 2021

Median incubation period^a^ *N* = 23, days (IQR^b^)	4 (2–8)
Inoculation eschar, no. (%)	35 (100)
Scalp	30 (86)
Trunk	2 (6)
Lower limbs	2 (6)
Brow bone	1 (3)
Lymphadenopathies, no. (%)	35 (100)
Only neck	26 (77)
Neck and other localization	7 (20)
Unique, *N* = 34	8 (23)
Multiple, *N* = 34	26 (76)
Supra-centimetric, *N* = 23	18 (76)
Painful, *N* = 23	17 (74)
Other symptoms, no. (%)	33 (94)
Fever	19 (54)
Headache	14 (40)
Asthenia	12 (34)
Rash	10 (29)
Arthromyalgia	6 (17)
Generalized rash	6 (17)
Neck pain	6 (17)
Alopecia	5 (14)
Facial edema	4 (11)
Chills	3 (9)
Median duration of symptoms^c^, days (IQR)	17 (11–27)

^a^Incubation period: time between the tick bite and the appearance of the first symptom

^*b*^*IQR* interquartile range

^c^Duration of symptoms: time between the appearance of the first symptom and the resolution of the last symptom

When *N* is not precise, the denominator is the total effective range (*N* = 35)

The inoculation eschar was located primarily on the scalp (86%) and was painful in 42% of the cases. Lymphadenopathies were mostly located in the neck but could be associated with other locations (inguinal, axillary, pretragal, or retroauricular).

Among other symptoms, fever was the most common, affecting more than half of the patients, often between 38° and 39 °C. Headache and asthenia were present in more than one-third of the patients, and rash was present in more than one-quarter. Six patients had generalized rash (one certain case with maculopapular exanthema, and among the five probable cases, two with maculopapular exanthema, one with urticaria, one with disseminated vesicles, and one with papulovesicular eruption with purpuric rash). Arthromyalgia, neck pain, facial edema, and alopecia were also observed.

Other symptoms, such as pruritus, scalp hypoesthesia, scalp pain or nodules, subcutaneous collection at the injection site, lymphangitis, nausea and vomiting, diarrhea, anorexia, altered general condition, cough, chest pain, and throat pain, were rarely observed. Two patients had complications of fistulation and suppuration of an inguinal lymphadenopathy. One patient had a *F. tularensis*-positive PCR for lymphadenopathy, and the other was a probable case. The latter required hospital care. No other patients were hospitalized. The median duration of symptoms was 17 days (IQR 11–27 days). One patient experienced asthenia for 6 months.

### Biological results

Complete blood counts (CBCs) were mostly normal, and the liver enzymes were not abnormal. C-reactive protein (CRP) levels were measured in 23 patients: the results were < 5 mg/L in 13 patients, moderately elevated (between 5 and 30 mg/L) in 5 patients, and elevated (> 30 mg/L) in 5 patients.

*Rickettsia* serology, which was performed in 27 patients, was positive in only 1 patient 10 days after the onset of symptoms. Among the two PCRs on biopsies performed on the inoculation eschar, one was positive for *R. slovaca*. Among the 12 *Rickettsia* PCRs performed on inoculation eschar swabs, 2 were positive.

Two *Dermacentor marginatus* ticks collected from patients were analyzed using microorganism-targeted PCR, one of which was positive for *R. slovaca*, *R. raoultii*, and *Coxiella-like* bacteria, while the other was positive for *Coxiella-like* bacteria (with negative *Coxiella burnetii*-specific PCR results).

Microbiological tests for *F. tularensis* (13 serologies, 5 PCRs on eschar swabs, 1 PCR on eschar biopsy, and 1 PCR on lymph node fluid) were performed: 2 serologies were positive, and 1 PCR on lymph node fluid was positive.

### Treatment

A total of 89% of patients received at least one effective treatment, but 34% of patients received two courses of antibiotics, and 14% received three or more (including local antibiotics such as fusidic acid). This led to inappropriate antibiotic therapy in 51% of the patients.

The median duration of antibiotic therapy was 15 days (IQR 7–19 days; data available for 30 patients). The median duration of effective antibiotic therapy was 14 days (IQR 6–15 days; data available for 27 patients). Table 3 presents the details of the nature and duration of treatment.

**Table 3:** Details of antibiotic therapy in patients retrospectively identified as TIBOLA in several hospitals in northeastern France between 2016 and 2021

Antibiotic treatment, *N* = 35	33
Doxycycline	26
Amoxicillin	13
Azithromycin	5
Amoxicillin–clavulanic acid	4
Topical fusidic acid	4
Pristinamycin	2
Ciprofloxacin	2
No treatment	2
Median duration, *N* = 30, days (IQR^a^)	15 (7–19)
Median duration with effective molecule^b^, *N* = 27 (IQR)	14 (6–15)
2 courses of antibiotics, %	34
> 3 courses of antibiotics, %	14
Exposure to inappropriate antibiotics, %	51
Exposure to beta-lactam antibiotics, %	46

^*a*^*IQR* interquartile range

^b^Effective molecules: doxycycline, azithromycin, and ciprofloxacin (if *F. tularensis* was identified as the causal agent)

When *N* is not precise, the denominator is the total effective range (*N* = 35)

### Dermacentor density

**Figure 5:**
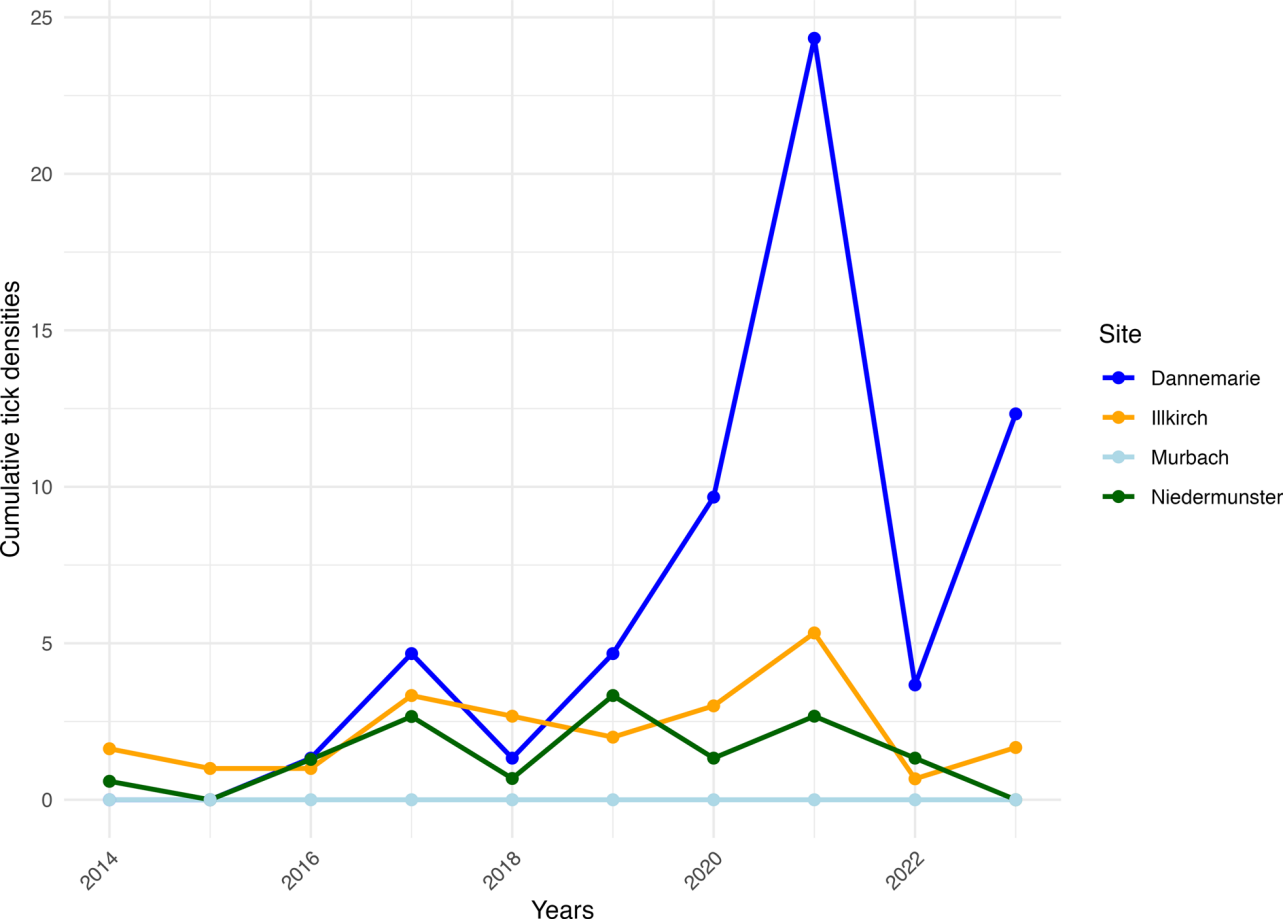
Longitudinal study of the cumulative *Dermacentor* density at four sites in the Alsace region from 2014 to 2023

The annual variation in the *Dermacentor* populations from four sites in the Alsace region was investigated at two mountainous sites (Murbach and Niedermunster) and two lowland sites (Illkirch and Dannemarie), as shown in Fig. 5. For Dannemarie, which is located in an agricultural area that produces corn, the *Dermacentor* population was particularly abundant in 2021. The two peaks of tick activity generally occurred from February to March and from October to November (data not shown).

## Discussion

This study highlights an increase in the number of TIBOLA cases in 2021 in northeastern France as well as an increase in the *Dermacentor* tick population. These results support the view that TIBOLA is a seasonal disease that occurs primarily in the spring [8]. TIBOLA patients are mainly female, young, and live in rural areas.

TIBOLA is a rare disease that is usually not reported in this region of France. Cases of TIBOLA also appear to have increased in other European countries, as shown in a recent Italian study [20]: the authors described ten cases on the basis of clinical and epidemiological criteria over a period of 7 years (2015–2022) in Tuscany, a region where TIBOLA was rarely found before.

Epidemiological changes also seem to occur in other tick-borne diseases. For example, tick-borne encephalitis, once found almost exclusively in a limited part of northeastern France, now seems to affect a wider area across the eastern half of France [12]. This increase in incidence has also been reported for tularemia by the European Center for Disease Prevention and Control [13, 14]. However, the incidence of Lyme borreliosis has been stable in France over the past few years [21, 22], and the incidence of Mediterranean spotted fever varies depending on the year [23].

The real challenge lies in the current lack of awareness and knowledge about TIBOLA among many physicians and its frequent confusion with Lyme disease [15], leading to inappropriate antibiotic therapy, such as amoxicillin. However, *Rickettsiae* are intracellular bacteria that are naturally resistant to beta-lactams [24]. One of the reasons why doxycycline has become the first-line treatment for Lyme borreliosis since 2018 is that it is effective against other tick-borne diseases [21]. This was confirmed by the 2025 guidelines [25].

There are few large-scale studies about TIBOLA in the scientific literature. Several case series, retrospective studies, and a few prospective studies can be found. Most of these studies are listed in Table 4. The seasonal fluctuations observed in this work, with the main peak in the spring and a smaller peak in the autumn, are consistent with those reported in previous studies [4, 16, 20, 26–28]. In this study, the median patient age was 32.5 years. In the literature, most patients diagnosed with TIBOLA are younger than 40 years old [3, 8, 29, 30]. Individuals who are minors represented a substantial proportion of the patients in the present study (34%), as observed in several other studies [4, 31]. There is no convincing pathophysiological hypothesis to explain why children are more affected by TIBOLA than adults are. The same predominance of children can be observed in Lyme borreliosis [32]. With respect to clinical features, 17% of patients reported a disseminated skin rash, which is far greater than what has been described in the literature: between 2% [8] and 5% of patients [30]. With respect to fever, the data in the literature are discordant: from 25% [8] to 80% of patients [20]. In the present study, half of the patients reported fever. Fewer cases of alopecia were reported here than in the literature: 14%, while previous studies reported 19% [8]. This finding could be explained by the retrospective design of this study, with a lack of standardized follow-up.

**Table 4:** Studies reporting cases of TIBOLA since 1997

Study	Location	Design	Inclusion criteria	No of cases	Season	Median age (years old)	% females	Microbiological documentation (*Rickettsia* spp.)
Lakos et al. 1997 [1]	Hungary	Retrospective, multicentric, 1 year	Tick bite and clinical criteria	27	Not mentioned	> 50% younger than 10		No microbiological confirmation
Lakos et al. 2002 [31]	Hungary	Prospective controlled, multicentric, 4 years	Tick bite or clinical criteria	86	March and April	13	72	Positive skin or lymph node PCR^a^: 10/13 Positive serology: 19/73
Raoult et al. 2002 [26]	France and Hungary	Retrospective, multicentric, 4 years	Tick bite and clinical criteria Definite case: positive culture or PCR	67	France: February to May Hungary: March and April	Unknown	61	17 microbiological confirmations: Positive serology: 7/17 Positive skin biopsy (PCR): 4/7 Positive lymph node biopsy: 7/8 Positive serum (PCR): 11/17 Positive tick (PCR): 3/3
Oteo et al. 2004 [30]	Spain	Retrospective-prospective, multicentric, 11 years	Tick bite by a large tick from October to April and clinical criteria and negative serology for *Borrelia burgdorferi* and *F. tularensis*	22	January, spring, and autumn (pick in November)	38	55	Positive serology: 9/12 Positive skin biopsy (PCR): 0/3
Gouriet et al. 2006 [29]	France	Retrospective, multicentric, 1 year	Tick bite and clinical criteria	14	October to January and February to May	35	64	Positive serology: 10/14 Positive skin biopsy (PCR): 3 Positive serum (PCR): 1 Positive tick (PCR): 3
Ibarra et al. 2006 [27]	Spain	Retrospective-prospective, multicentric, 14 years	Tick bite by a large tick from October to May and clinical criteria	54	April and November	37	59	Positive serology: 19/31 Positive serum (PCR): 9/21 Positive skin biopsy (PCR): 0 Positive tick (PCR): 10/10
Porta et al. 2008 [37]	Spain	Retrospective, monocentric, 6 years	Tick bite and clinical criteria	36	October to April	16	44	Positive serology: 10 Positive PCR on skin, serum, and eschar swab: 0 Positive tick (PCR): 4/7
Selmi et al. 2008 [28]	Italy	Prospective, multicentric, 2 years	Tick bite and clinical criteria, consulting the emergency department to remove the tick	5	Spring		60	Positive tick (PCR): 3/5
Parola et al. 2009 [3]	France	Retrospective, multicentric, 6 years	Tick bite and serum/skin biopsy/tick received at the laboratory or clinical criteria Certain case: culture or PCR positive on patient sample Probable case: serology or positive PCR on tick	86	February to May	32	71 (among certain cases)	56 microbiological confirmations: Positive serology: 66/78 Positive tick (PCR): 13/18 Positive skin biopsy (PCR): 4/19 Positive skin biopsy (culture): 3/19 Positive serum (PCR): 1
Lakos et al. 2012 [38]	Hungary	Retrospective (case control study), multicentric, 3 years	Tick bite and clinical criteria	50	May to November	9	82	Positive serology: 23/47
Beytout et al. 2013 [39]	France	Retrospective, monocentric,15 years	Tick bite and/or clinical criteria	17				Positive serology: 6/14 Positive skin biopsy (PCR): 1
Rigal et al. 2014 [40]	France	Series of cases, monocentric, 10 months	Tick bite and clinical criteria	5	November and June		100	Positive serology: 1
Dubourg et al. 2014 [16]	France	Prospective, multicentric, 5 years	Tick bite and clinical criteria Definite case: positive culture or PCR or serology	56	Spring and autumn	42	68	Positive serology: 2/45 Positive skin biopsy (PCR): 3/16 Positive eschar swab or crust (PCR): 6/34 Tick positive (PCR): 7/9 Positive culture (tick, skin biopsy, or eschar swab): 4
Silva-Pinto et al. 2014 [8]	International	Literature review, multicentric, 16 years	Not standardized	37 articles 537 patients	Spring, autumn and winter	Always younger than 40	64	Microbiological confirmation in 149 cases
Santibañez et al. 2022 [4]	Spain	Retrospective, multicentric, 19 years	Clinical and epidemiological criteria	216	November, April, and May	40	65	Positive serology: 91/109 Positive serum (PCR): 14/104 Positive skin biopsy (PCR): 7/7 Positive eschar swab (PCR): 69/142 Positive tick (PCR): 71/71
Barbiero et al. 2023 [20]	Italy	Retrospective, monocentric, 7 years	Tick bite and clinical criteria	10	March–May	46	100	No microbiological confirmation Positive serology: 0/10 Positive skin biopsy (PCR): 0/2 Positive serum (PCR): 0/5 Positive eschar swab (PCR): 0/4
Present study	France	Retrospective, multicentric, 6 years	Tick bite and clinical criteria Certain case if positive PCR or serology, probable case if not	35	April and May	33	83	Positive serology: 1/19 Positive skin biopsy (PCR): 1/2 Positive eschar swab (PCR): 2/12 Positive tick (PCR): 1/2

^a^*PCR* polymerase chain reaction

TIBOLA diagnostic criteria are not well defined. Some studies are based on clinical signs following a tick bite [27], whereas others require microbiological documentation [3]. In the present study, certain and probable cases of TIBOLA were distinguished. In most cases, a TIBOLA diagnosis is based on a combination of demographic, epidemiological, and clinical factors. Microbiological documentation is particularly difficult and frequently not essential, especially in primary care units, where biological analyses are usually not performed. The absence of microbiological documentation is frequent in the literature, as was the case in the present study. Studies performed in National Reference Centers (Marseille in France, La Rioja in Spain), which routinely perform specific *Rickettsia* serology and PCR, have a higher microbiological documentation rate [3, 4, 26, 27, 30] since they include referred patients with a higher probability of TIBOLA.

TIBOLA is a local or regional disease with an inconsistent blood phase; this may explain why the serology is often negative, with a sensitivity of 12% [26]. Furthermore, recommendations suggest that serological tests should be repeated to observe seroconversion, which was rarely performed in the present study [17]. In addition, *R. raoultii* is difficult to identify in humans. Some studies suggest that *D. reticulatus* primarily harbors *R. raoultii* and that *D. marginatus* harbors *R. slovaca* [10, 33]. *Dermacentor reticulatus* is predominant in northeastern France [8]; this could explain the low proportion of microbiologically confirmed cases in this study.

A question arises as to the factors determining the possible expansion of TIBOLA. Global warming could be one of the causes of this increase via a change in the geographical and seasonal distribution of *Dermacentor* ticks, as evidenced by the detection of this tick as early as January [34, 35]. Global warming could also induce behavioral changes in hosts, including humans, increasing their outdoor activities and potentially their exposure to arthropod bites. Human activities impacting tick biotopes are also likely to favor vector expansion [18] through the evolution of agriculture over recent decades, through the reforestation and importation of animals, and through a reduction in the use of pesticides (even if this behavior change could have other advantages for human health). In Germany, a participatory citizen study (ticks sent by citizens, collection of geographical information) performed from 2020–2021 revealed the spatial spread of *Dermacentor* [36]. Entomological studies are currently being conducted to document the spread of *Dermacentor* ticks in nature and to explain the determining factors (*Borrelia* National Reference Center, Strasbourg, France). Preliminary data show that in northeastern France, 19% of *D. reticulatus* are infected by *R. raoultii*, whereas *R. slovaca* is rarely detected (1%). Interannual variations rather than real increases in *Dermacentor* were observed, and these ticks are present in specific ecosystems (N. Boulanger, personal communication).

Furthermore, a greater number of TIBOLA might be not only a consequence of an emergence, but also of a better awareness of tick-borne diseases by medical professionals in France.

This study combining human and tick epidemiological data is original but has several limitations linked to its retrospective design, particularly missing data and memory bias among clinicians. To control these biases, microbiological data were extracted to identify cases that the clinicians could not remember. Wide screening based on prescribed tests and careful examination of medical files helped to contain them. This strategy led to the inclusion of several types of tularemia. This disease matches the predefined inclusion criteria: inoculation eschar with regional lymphadenopathy after a tick bite. Hence, a question arises about whether TIBOLA is a disease or a syndrome.

TIBOLA is benign and usually has no systemic complications. In most cases, patients do not seek specialized hospital care. This study estimated the number of cases of TIBOLA diagnosed in hospitals in northeastern France, but most of the cases were probably met in primary care, resulting in selection bias. Thus, the number of cases must have been underestimated. The inclusion of outpatients is methodologically and financially complex, and most of the time, patients seen in primary care units do not have microbiological tests. Thus, in an outpatient study, the microbiological documentation rate would have been even lower. Another limitation is that the screening was performed in a limited number of hospitals. In addition, two cases were diagnosed in northeastern France after a tick bite in another region. In terms of entomological data, there were only four collection sites in Alsace, a region that is nevertheless endemic for ticks and tick-borne diseases, whereas the clinical inclusion zone was wider in northeastern France. Work is in progress to complete vector epidemiology.

## Conclusions

This study confirms that TIBOLA is a benign, rare, and difficult-to-diagnose disease. These results suggest that TIBOLA could become an emerging disease in northeastern France if environmental conditions favor the development of the vector and its contact with humans.

## Data Availability

Data supporting the main conclusions of this study are included in the manuscript.

## Abbreviations

CBC: Complete blood count
°C: Degrees Celsius
COI: Cytochrome oxidase I gene
CRP: C-reactive protein
DEBONEL: *Dermacentor* Borne necrosis erythema and lymphadenopathy
IgG: Immunoglobulin G
IgM: Immunoglobulin M
IQR: Interquartile range
MALDI-TOF: Matrix-assisted laser desorption ionization time of flight
mg/L: Milligrams per liter
PCR: Polymerase chain reaction
rDNA: Recombinant desoxyribonucleic acid
SENLAT: Scalp eschar and neck lymphadenopathy after tick bite
TIBOLA: Tick borne lymphadenopathy

## References

[1] Lakos A. TIBOLA-a new tick-borne infection. Orv Hetil. 1997;138:3229–32. 9454101

[2] Sekeyová Z, Danchenko M, Filipčík P, Fournier PE. Rickettsial infections of the central nervous system. PLoS Negl Trop Dis. 2019;13:e0007469. 31465452 10.1371/journal.pntd.0007469 PMC6715168

[3] Parola P, Rovery C, Rolain JM, Brouqui P, Davoust B, Raoult D. *Rickettsia**slovaca* and *R. raoultii* in tick-borne rickettsioses. Emerg Infect Dis. 2009;15:1105–8. 19624931 10.3201/eid1507.081449 PMC2744242

[4] Santibáñez S, Portillo A, Ibarra V, Santibáñez P, Metola L, García-García C et al. Epidemiological, clinical, and microbiological characteristics in a large series of patients affected by *Dermacentor*-borne-necrosis-erythema-lymphadenopathy from a unique centre from Spain. Pathogens. 2022;11:528. 35631049 10.3390/pathogens11050528 PMC9146834

[5] Angelakis E, Pulcini C, Waton J, Imbert P, Socolovschi C, Edouard S et al. Scalp eschar and neck lymphadenopathy caused by *Bartonella henselae* after tick bite. Clin Infect Dis. 2010;50:549–51. 20070235 10.1086/650172

[6] Cascio A, Torina A, Valenzise M, Blanda V, Camarda N, Bombaci S et al. Scalp eschar and neck lymphadenopathy caused by *Rickettsia**massiliae*. Emerg Infect Dis. 2013;19:836. 10.3201/eid1905.121169 PMC3647502 23697545

[7] Edouard S, Gonin K, Turc Y, Angelakis E, Socolovschi C, Raoult D. Eschar and neck lymphadenopathy caused by *Francisella**tularensis* after a tick bite: a case report. J Med Case Rep. 2011;5:108. 21418587 10.1186/1752-1947-5-108 PMC3069950

[8] Silva-Pinto A, de Lurdes SM, Sarmento A. Tick-borne lymphadenopathy, an emerging disease. Ticks Tick-Borne Dis. 2014;5:656–9. 25090977 10.1016/j.ttbdis.2014.04.016

[9] Boucher D, Guimard T. Étude des syndromes escarre-ganglion(s) ou « TIBOLA » (Tick-BOrne LymphAdenopathy) sur un recueil réalisé au Centre Hospitalier Départemental Vendée de 2010 à 2016. 2017. https://books.google.fr/books?id=Us1tzgEACAAJ. Accessed 9 July 2023.

[10] Buczek W, Koman-Iżko A, Buczek AM, Buczek A, Bartosik K, Kulina D et al. Spotted fever group *rickettsiae* transmitted by *Dermacentor* ticks and determinants of their spread in Europe. Ann Agric Environ Med. 2020. 10.26444/aaem/120602. 33356053 10.26444/aaem/120602

[11] Raoult Didier. Les rickettsioses/Didier Raoult et Philippe Brouqui ; avec la collab. de Michel Drancourt, Pierre Edouard Fournier, Pierre Houpikian et al. 1998. Elsevier. Paris.

[12] SPF. Encéphalites à tiques (TBE) en France. Bilan des cas déclarés de mai 2021 à mai 2023. https://www.santepubliquefrance.fr/maladies-et-traumatismes/maladies-a-transmission-vectorielle/encephalite-a-tiques/documents/encephalites-a-tiques-tbe-en-france.-bilan-des-cas-declares-de-mai-2021-a-mai-2023. Accessed 8 Aug 2023

[13] Tularemie - Donnees epidemiologiques. 2018 [Internet]. [cited 2024 Jan 28]. Available from: https://www.santepubliquefrance.fr/maladies-et-traumatismes/maladies-transmissibles-de-l-animal-a-l-homme/tularemie/documents/donnees/tularemie-donnees-epidemiologiques-2018.Accessed 28 Jan 2024.

[14] European Food Safety Authority, European Centre for Disease Prevention and Control. The European Union One Health 2021 Zoonoses Report. EFS2. 2022. https://data.europa.eu/doi/10.2903/j.efsa.2022.7666. Accessed 28 Jan 2024. 10.2903/j.efsa.2022.7666 PMC9745727 36524203

[15] Foissac M, Socolovschi C, Raoult D. Les nouveautés sur le syndrome SENLAT : Scalp Eschar and Neck LymphAdenopathy after Tick bite. Ann Dermatol Venereol. 2013;140:598–609. 24090889 10.1016/j.annder.2013.07.014

[16] Dubourg G, Socolovschi C, Del Giudice P, Fournier PE, Raoult D. Scalp eschar and neck lymphadenopathy after tick bite: an emerging syndrome with multiple causes. Eur J Clin Microbiol Infect Dis. 2014;33:1449–56. 24682865 10.1007/s10096-014-2090-2

[17] Borréliose de Lyme et autres maladies vectorielles à tiques. Haute Autorité de Santé. 2023. https://www.has-sante.fr/jcms/c_2857558/fr/borreliose-de-lyme-et-autres-maladies-vectorielles-a-tiques. Accessed 7 Aug 2023.

[18] Boyer PH, Barthel C, Mohseni-Zadeh M, Talagrand-Reboul E, Frickert M, Jaulhac B et al. Impact of different anthropogenic environments on ticks and tick-associated pathogens in Alsace, a French region highly endemic for tick-borne diseases. Microorganisms. 2022;10:245. 35208700 10.3390/microorganisms10020245 PMC8877010

[19] Boyer PH, Almeras L, Plantard O, Grillon A, Talagrand-Reboul É, McCoy K et al. Identification of closely related *Ixodes* species by protein profiling with MALDI-TOF mass spectrometry. PLoS ONE. 2019;14:e0223735. 31622384 10.1371/journal.pone.0223735 PMC6797106

[20] Barbiero A, Manciulli T, Spinicci M, Vellere I, Colao MG, Rossolini GM et al. Scalp eschar and neck lymph adenopathy after a tick bite (SENLAT) in Tuscany, Italy (2015–2022). Infection. 2023. 10.1007/s15010-023-02079-8. 37563481 10.1007/s15010-023-02079-8 PMC10665257

[21] Raffetin A, Hansmann Y, Sauvat L, Schramm F, Tattevin P, Baux E et al. Borreliose de Lyme. Rev Prat. 2023;73:187–96. 36916263

[22] Université réseau S INSERM/Sorbonne. Réseau Sentinelles > France >. 2024. https://www.sentiweb.fr/france/fr/?page=table&maladie=18.Accessed 14 Dec 2024.

[23] Spernovasilis N, Markaki I, Papadakis M, Mazonakis N, Ierodiakonou D. Mediterranean spotted fever: current knowledge and recent advances. Trop Med Infect Dis. 2021;6:172. 34698275 10.3390/tropicalmed6040172 PMC8544691

[24] Hocquart M, Drouet H, Levet P, Raoult D, Parola P, Eldin C. Cellulitis of the face associated with SENLAT caused by *Rickettsia**slovaca* detected by qPCR on scalp eschar swab sample: an unusual case report and review of literature. Ticks Tick-borne Dis. 2019;10:1142–5. 31213411 10.1016/j.ttbdis.2019.06.010

[25] Borréliose de Lyme et autres maladies vectorielles à tiques (MVT). Haute Autorité de Santé. 2025. https://www.has-sante.fr/jcms/c_2857558/fr/borreliose-de-lyme-et-autres-maladies-vectorielles-a-tiques-mvt. Accessed 19 Apr 2025.

[26] Raoult D, Lakos A, Fenollar F, Beytout J, Brouqui P, Fournier P. Spotless rickettsiosis caused by *Rickettsia**slovaca* and associated with *Dermacentor* ticks. Clin Infect Dis. 2002;34:1331–6. 11981728 10.1086/340100

[27] Ibarra V, Oteo JA, Portillo A, Santibanez S, Blanco JR, Metola L, Eiros JM, Perez Martinez L, Sanz M. *Rickettsia**slovaca* infection: DEBONEL/TIBOLA. Ann N Y Acad Sci. 2006;1078:206–14. 10.1196/annals.1374.040 17114711

[28] Selmi M, Bertolotti L, Tomassone L, Mannelli A. *Rickettsia**slovaca* in *Dermacentor**marginatus* and tick-borne lymphadenopathy, Tuscany, Italy. Emerg Infect Dis. 2008;14:817. 18439371 10.3201/eid1405.070976 PMC2600248

[29] Gouriet F, Rolain JM, Raoult D. *Rickettsia**slovaca* Infection, France. Emerg Infect Dis. 2006;12:521. 16710981 10.3201/eid1203.050911 PMC3293430

[30] Oteo JA, Ibarra V, Blanco JR, Martínez de Artola V, Márquez FJ, Portillo A, Raoult D, Anda P. *Dermacentor*-borne necrosis erythema and lymphadenopathy: clinical and epidemiological features of a new tick-borne disease. Clin Microbiol Infect. 2004;10:327–31. 10.1111/j.1198-743X.2004.00782.x 15059122

[31] Lakos A. Tick-borne lymphadenopathy (TIBOLA). Wien Klin Wochenschr. 2002;114:648–54. 12422619

[32] Borréliose de Lyme : données épidémiologiques 2020. https://www.santepubliquefrance.fr/les-actualites/2021/borreliose-de-lyme-donnees-epidemiologiques-2020. Accessed 14 Dec 2024.

[33] Špitalská E, Štefanidesová K, Kocianová E, Boldiš V. *Rickettsia**slovaca* and *Rickettsia**raoultii* in *Dermacentor**marginatus* and *Dermacentor**reticulatus* ticks from Slovak Republic. Exp Appl Acarol. 2012;57:189–97. 22392435 10.1007/s10493-012-9539-8

[34] Koczwarska J, Pawełczyk A, Dunaj-Małyszko J, Polaczyk J, Welc-Falęciak R. *Rickettsia* species in *Dermacentor**reticulatus* ticks feeding on human skin and clinical manifestations of tick-borne infections after tick bite. Sci Rep. 2023;13:9930. 37336983 10.1038/s41598-023-37059-3 PMC10279655

[35] Földvári G, Široký P, Szekeres S, Majoros G, Sprong H. *Dermacentor**reticulatus*: a vector on the rise. Parasit Vectors. 2016;9:314. 27251148 10.1186/s13071-016-1599-x PMC4888597

[36] Springer A, Lindau A, Probst J, Drehmann M, Fachet K, Thoma D, Rose Vineer H, Noll M, Dobler G, Mackenstedt U, Strube C. Update and prognosis of *Dermacentor* distribution in Germany: nationwide occurrence of *Dermacentor**reticulatus*. Front Vet Sci. 2022;9:1044597. 10.3389/fvets.2022.1044597 PMC9666490 36406070

[37] Porta FS, Nieto EA, Creus BF, Espín TM, Casanova FJT, Sala IS et al. Tick-borne lymphadenopathy: a new infectious disease in children. Pediatr Infect Dis J. 2008;27:618–22. 18520970 10.1097/INF.0b013e31816b1947

[38] Lakos A, Kőrösi Á, Földvári G. Contact with horses is a risk factor for tick-borne lymphadenopathy (TIBOLA): a case control study. Wien Klin Wochenschr. 2012;124:611–7. 22878792 10.1007/s00508-012-0217-y

[39] Beytout J, Romaszko J-P, Vidal M, Gourdon F. Tick born lymphadenitis (TIBOLA) : 17 observations d’un service d’infectiologie. 2024. https://www.infectiologie.com/UserFiles/File/medias/JNI/JNI13/posters/2013-JNI-F-10.pdf. Accessed 14 Dec 2024.

[40] Rigal E, Dorcier D, Lesens O, Texier C, D’Incan M. TIBOLA : une rickettsiose en expansion, cliniquement polymorphe. Ann Dermatol Venereol. 2014;141:186–91. 24635952 10.1016/j.annder.2013.11.001

